# Patellar resurfacing versus patellar nonresurfacing in primary total knee arthroplasty: A protocol for systematic review and meta analysis

**DOI:** 10.1097/MD.0000000000020097

**Published:** 2020-05-22

**Authors:** Chengzhi Hou, Xuelei Chu, Bingbing Zhang, Jiaxian Li, Yongli Dong, Yong Zhao

**Affiliations:** a Orthopedic Department, Wangjing Hospital, China Academy of Chinese Medical Sciences; b Beijing University of Chinese Medicine; c Eye Hospital, China Academy of Chinese Medical Sciences, Beijing, China.

**Keywords:** meta-analysis, nonresurfacing, patellar resurfacing, primary total knee arthroplasty, protocol

## Abstract

**Background:**

Total knee arthroplasty (TKA) is one of the most common orthopedic procedures. However, the decision to resurface the patella during a primary TKA remains controversial. Therefore, a systematic review and meta-analysis were conducted to determine whether patellar resurfacing is needed in primary total knee arthroplasty.

**Methods:**

A systematic literature research will be conducted in 7 databases including PubMed, Embase, Cochrane Library website, ClinicalTrials.gov databases, Chinese National Knowledge Infrastructure Database, Wanfang database, and VIP database for Chinese Technical Periodicals. The quality of studies will be assessed according to Cochrane risk of bias tool and Methodological index for non-randomized studies (MINORS) scale. The level of the evidence will be estimated by grading of recommendations assessment, development, and evaluation system. Data analysis and synthesis will be completed by the Review Manager 5.3.

**Conclusions:**

The conclusion of this study will provide clinicians performing TKA with a recommendation whether to conduct patellar resurfacing and further guide the clinical decision-making.

PROSPERO registration number: CRD42019129711.

## 1 Introduction

Total knee arthroplasty (TKA), one of the most common orthopedic procedures, is used to correct deformity, alleviate pain, and restore joint function.^[1]^ Due to feasible and cost-effective, the number of TKA continues to increase year after year.^[2]^ At present, the decision to resurface the patella during a primary TKA remains controversial.^[3]^ There are 3 different strategies as follows: patellar resurfacing, nonresurfacing, and selective resurfacing depending on various patient, radiographic change, and clinical criteria.^[4,5]^ According to the epidemiological survey,^[6]^ 82% of TKA patients have performed patellar resurfacing in the United States, while only 2% in Norway and Sweden. Besides, there are more than 50% of TKA performed patellar resurfacing in the Netherlands and the United Kingdom.

Anterior knee pain is one of the most common complications after TKA.^[7]^ Studies have reported patellar resurfacing played an essential role in reducing postoperative anterior knee pain, reoperation rate, and improving knee function.^[8,9]^ In addition, patellar resurfacing is cost-effective because nonresurfacing have higher revision rates.^[10]^ However, conducting patellar resurfacing in primary TKA also leads to increase of complication rates and complications commonly seen with patellar resurfacing include patella fractures, avascular necrosis, overstuffing, patellofemoral instability, and even component wear dissociation or loosening.^[3,8,11]^

At present, there are many randomized controlled trials (RCTs) and meta-analyses on the comparison between patellar resurfacing and nonresurfacing primary TKA,^[8,9,11–15]^ but most of which only focusing on postoperative functional comparison and failing to provide the systematic and comprehensive understanding of patellar resurfacing for us. Many problems also need to be considered in the research such as whether the clinical effect affected by different patients, whether the outcome of patellar resurfacing affected by different patellar treatments, whether the effect of early postoperative is the same as the late effect. Therefore, the purpose of this study is to provide clinicians performing TKA with a synthesis of evidence whether to conduct patellar resurfacing and further guide the clinical decision-making. Meanwhile, This evidence helps clinicians understand more comprehensively and systematically patellar resurfacing.

## 2 Methods

### 2.1 Protocol and registration

This is a literature-based study, thus ethical approval are not required. The protocol of this study follows the guideline of the preferred reporting items for systematic reviews and meta-analyses protocols^[16]^ and has been registered on the International Prospective Register of Systematic Reviews with CRD42019129711.

### 2.2 Searching strategy

Two independent reviewers will search for databases including PubMed, Embase, Cochrane Library website, ClinicalTrials.gov databases, Chinese National Knowledge Infrastructure Database, Wanfang database, and VIP database using the search strategies recommended by the Cochrane Back Review Group. There is no limit on the language and publication type. Meanwhile, the date of searching literature published before February 1, 2020. The English search terms included following: “total knee arthroplasty,” “patellar resurfacing,” and “trial” with the Boolean logic operator “AND” and “OR.” Furthermore, reference cited in the relevant literature and other articles in the meta-analysis will be also reviewed.

### 2.3 Inclusion and exclusion criteria

Studies will be examined whether meets the following criteria.

(1) The type of study is RCT or clinical control trial.
(2) Patients are suffered from primary TKA.
(3) Clinical main outcomes include anterior knee pain, knee society score, knee society functional score, Western Ontario and McMaster Universities score, complication and reoperation, satisfaction.
(4) The included studies must contain patellar resurfacing group and nonresurfacing group.

Relatively, studies will be excluded if can not meet the inclusion criteria.

### 2.4 Data extraction

To avoid bias from the data extraction process, all data will be extracted independently by 2 reviewers, result of which will be compared. The characteristic information extracted from each study included the following: name of first author, year of publication, research type, country, sample size, prosthesis type, patellar treatments, outcome measures, follow-up duration, age, gender, disease, and surgical method of patients. If opinion of 2 reviewers is different, the third reviewer will join in the discussion and make a suggestion.

### 2.5 Quality assessment

The quality of RCT studies will be assessed according to Cochrane risk of bias tool.^[17]^ The result of assessment include following 6 aspects: generation of random sequence; concealment for allocation sequence; application of blinding; incomplete outcome data; selective outcome reporting, and other bias. According to result of assessment, the risk types of bias are divided into “high,” “unclear,” and “low.” The quality of clinical control trial studies will be assessed using MINORS scale.^[18]^ The risk of bias will be examined by 2 reviewers at the same time, and decision will be made by consensus.

### 2.6 Statistical methods

The RevMan 5.3 software (Cochrane Collaboration, Oxford, UK) will be used to conduct the meta-analyses. The difference of continuous variables in each study will be estimated using mean difference. The standardized mean difference will be used if continuous variables are large or are expressed using different units. The risk ratio and the corresponding 95% confidence interval will be used for the dichotomous variables. Homogeneity of included studies was determined by the *I*-squared statistic (*I*^2^).^[19]^ The fixed-effects model will be used to conduct a meta-analysis if the heterogeneity is low (*I*^2^ < 50%). Otherwise, if heterogeneity is significant (*I*^2^ > 50%), the random-effects will be used.^[20]^ Sensitivity analyses will be evaluated by removing studies with high risk of bias or excluding one-by-one. Publication bias was evaluated by funnel plots and Egger test.

### 2.7 Grading of recommendations assessment, development, and evaluation

The level of the evidence will be estimated by the grading of recommendations assessment, development, and evaluation system. There are 4 levels: high, moderate, low, and very low during the assessment of quality.

## 3 Discussion

The number of TKA have grown rapidly, however, it is still controversial whether patellar resurfacing is needed in primary TKA. This protocol presents the methodology of a systematic review and meta-analysis for determining whether or not patellar resurfacing is a superior method for patients in primary TKA than nonresurfacing. Furthermore, according to the preferred reporting items for systematic reviews and meta-analyses protocols, the result of this review is systematically and comprehensively assessed and expects to provide more reliable evidence for clinicians performing TKA.

## Author contributions

**Conceptualization:** Chengzhi Hou.

**Data curation:** Chengzhi Hou, Bingbing Zhang.

**Formal analysis:** Chengzhi Hou, Xuelei Chu.

**Funding acquisition:** Yong Zhao.

**Methodology:** Chengzhi Hou, Jiaxian Li.

**Resources:** Chengzhi Hou, Yongli Dong.

**Software:** Bingbing Zhang.

**Supervision:** Yong Zhao.

**Writing original draft:** Chengzhi Hou, Xuelei Chu.

**Writing review and editing:** Chengzhi Hou, Xuelei Chu, Yongli Dong, Yong Zhao.
